# Eliminating Risk of Intubation in Very Preterm Infants with Noninvasive Cardiorespiratory Support in the Delivery Room and Neonatal Intensive Care Unit

**DOI:** 10.1155/2019/5984305

**Published:** 2019-01-13

**Authors:** Balaji Govindaswami, Matthew Nudelman, Sudha Rani Narasimhan, Angela Huang, Sonya Misra, Gilbert Urquidez, Alganesh Kifle, Monica Stemmle, Cathy Angell, Rupalee Patel, Christina Anderson, Dongli Song, Glenn DeSandre, James Byrne, Priya Jegatheesan

**Affiliations:** ^1^Santa Clara Valley Medical Center: Hospitals and Clinics, Department of Pediatrics, Newborn Medicine, San Jose, CA, USA; ^2^Stanford University School of Medicine, Stanford, CA, USA; ^3^San Jose State University School of Nursing, San Jose, CA, USA; ^4^Santa Clara Valley Medical Center: Hospitals and Clinics, Department of Obstetrics and Gynecology, Maternal-Fetal Medicine, San Jose, CA, USA

## Abstract

**Introduction:**

Avoiding intubation and promoting noninvasive modes of ventilator support including continuous positive airway pressure (CPAP) in preterm infants minimizes lung injury and optimizes neonatal outcomes. Discharge home on oxygen is an expensive morbidity in very preterm infants (VPI) with lung disease. In 2007 a standardized bundle was introduced for VPI admitted to the neonatal care unit (NICU) which included delayed cord clamping (DCC) at birth and noninvasive ventilation as first-line cardiorespiratory support in the delivery room (DR), followed by bubble CPAP upon NICU admission.

**Objective:**

Our goal was to evaluate the risk of (1) intubation and (2) discharge home on oxygen after adopting this standardized DR bundle in VPI born at a regional perinatal center and treated in the NICU over a ten-year period (2008-2017).

**Materials and Methods:**

We compared maternal and neonatal demographics, respiratory care processes and outcomes, as well as neonatal mortality and morbidity in VPI (< 33 weeks gestation) and extremely low birth weight (ELBW, < 1000 g) subgroup for three consecutive epochs: 2008-2010, 2011-2013, and 2014-2017.

**Results:**

Of 640 consecutive inborn VPI, 55% were < 1500 g at birth and 23% were ELBW. Constant through all three epochs, DCC occurred in 83% of VPI at birth. There was progressive increase in maternal magnesium during the three epochs and decrease in maternal antibiotics during the last epoch. Over the three epochs, VPI had less risk of DR intubation (23% versus 15% versus 5%), NICU intubation (39% versus 31% versus 18%), and invasive ventilation (37% versus 30% versus 17%), as did ELBW infants. Decrease in postnatal steroid use, antibiotic exposure, and increase in early colostrum exposure occurred over the three epochs both in VPI and in ELBW infants. There was a sustained decrease in surfactant use in the second and third epochs. There was no significant change in mortality or any morbidity in VPI; however, there was a significant decrease in pneumothorax (17% versus 0%) and increase in survival without major morbidity (15% versus 41%) in ELBW infants between 2008-2010 and 2014-2017. Benchmarked risk-adjusted rate for oxygen at discharge in a subgroup of inborn VPI (401-1500 g or 22-31 weeks of gestation) is 2.5% (2013-2017) in our NICU compared with > 8% in all California NICUs and > 10% in all California regional NICUs (2014-2016).

**Conclusion:**

Noninvasive strategies in DR and NICU minimize risk of intubation in VPI without adversely affecting other neonatal or respiratory outcomes. Risk-adjusted rates for discharge home on oxygen remained significantly lower for inborn VPI compared with rates at regional NICUs in California. Reducing intubation risk in ELBW infants may confer an advantage for survival without major morbidity. Prenatal magnesium may reduce intubation risk in ELBW infants.

## 1. Introduction

Intubation risk in the delivery room (DR) for very preterm (< 33 weeks gestational age [GA]) infants (VPI) has declined over the last decade as early continuous positive airway pressure (CPAP) has gained increasing acceptance [1].

Improved perinatal practice, inclusive of antenatal steroid (ANS) use enhancing fetal lung maturity, has improved infants' ability to establish effective spontaneous ventilation at birth. At the same time, use of prenatal magnesium sulfate (a musculoskeletal depressant) for infant neuroprotection has been encouraged. DR CPAP assists infant cardiopulmonary transition at birth. Prenatally, the lung is filled with fluid that maintains functional residual capacity (FRC). At birth, this fluid is present in alveoli and is rapidly absorbed into the interstitial spaces, leading to transient respiratory dysfunction. In the preterm infant, transition is more difficult. FRC may be lost as VPI have higher frequency of caesarean section (CS), associated with increased lung fluid at delivery and lower serum protein, leading to slower resolution of interstitial edema. Inadequate surfactant production, weak muscles, and compliant chest wall also predispose VPI to atelectasis [2]. CPAP reduces lung injury by preventing/reducing atelectasis [3] and enhances fetal lung cell growth by mimicking the back pressure of lung fluid [4]. CPAP is used in the neonatal intensive care unit (NICU) to enhance cardiopulmonary development by optimizing FRC and reducing work of breathing for the baby.

**Figure 1:**
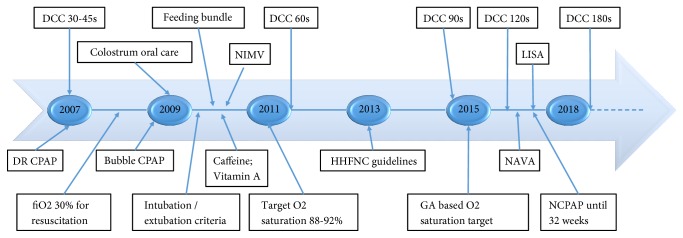
Timeline of introduction of standardized practice changes. DCC = delayed cord clamping; DR CPAP = delivery room continuous positive airway pressure; NIMV = noninvasive mechanical ventilation; HHFNC = humidified high-flow nasal cannula; NAVA = neurally adjusted ventilatory assist; LISA = less invasive surfactant administration.

In mid-2007, we introduced delayed cord clamping (DCC) for VPI and noninvasive ventilation as first-line cardiorespiratory support in the DR, followed by bubble CPAP on admission to NICU. We have subsequently shown reduced DR and NICU intubation risk associated with this practice [5, 6]. Other practice changes for VPI have occurred to lower NICU comorbidity (lowering risk of subsequent intubation), including buccal colostrum administration as part of ventilator-associated pneumonia (VAP) prophylaxis in 2009 (Figure 1). It is known that mortality is reduced in regional centers with availability of a wide range of requisite subspecialists [7] and that discharge home on oxygen requires complex follow-up [8]. A United States National Institute of Child Health and Human Development (NICHD) study of 9,575 infants 22-28 weeks GA and birth weight 401-1500 g born 2003-2007 [9] noted that rates of DR intubation and surfactant therapy varied by GA and that these rates had decreased temporally for infants born at 28 weeks GA but not for those < 28 weeks GA. Our goal is to evaluate the risk of intubation, frequency and duration of invasive and noninvasive ventilation, and discharge home on oxygen in VPI in the era of DR CPAP, while describing temporal trends over a decade (2008-2017).

## 2. Materials and Methods

This retrospective cohort study was IRB approved as a quality improvement project. We examined 640 consecutive inborn VPI, born at < 33 weeks of gestation from 2008-2017 and admitted to our NICU located in San Jose, CA. Our perinatal regional center includes one of 23 California regional NICUs (18 of which have inborn deliveries) with 3000-5000 live births per annum during the study period. Our VPI rate during this period was 1.6 per 100 live births. We compared three consecutive epochs: 2008-2010 (Epoch 1), 2011-2013 (Epoch 2), and 2014-2017 (Epoch 3).

During the study period, various aspects of VPI care were standardized from DR management, initial respiratory support on admission, and throughout NICU stay. Figure 1 shows practice changes over the last decade relevant to DR and respiratory care pertinent to VPI, inclusive of standardized care to decrease NICU comorbidity.

Maternal demographics, as well as neonatal demographics, interventions, and outcomes were obtained from the NICU database. These included birth weight, GA, sex, mode of delivery, ANS, perinatal magnesium, maternal and neonatal antibiotic exposure, neonatal medications (caffeine, vitamin A, postnatal steroids, surfactant), respiratory support measures both as exposure and duration in days (DR intubation, invasive mechanical ventilation, noninvasive mechanical ventilation [NIMV], CPAP, nasal cannula), respiratory outcomes (NICU intubation, pneumothorax, physiological chronic lung disease (CLD) at 36 weeks postmenstrual age [PMA], discharge on oxygen), NICU length of stay, PMA at discharge, mortality, and morbidities (severe intraventricular hemorrhage [grade 3 or 4], severe retinopathy of prematurity [stage 3 or 4, or plus disease or received intraocular Bevacizumab treatment], necrotizing enterocolitis [NEC] Bell's stage 2 or greater, spontaneous intestinal perforation, nosocomial infection [blood or cerebrospinal fluid culture positive at > 72 hours of life]). Exposure to invasive ventilation was defined as an infant who received conventional mechanical ventilation or high frequency ventilation in the NICU. NICU intubation was defined as endotracheal tube placement for mechanical ventilation or for surfactant administration without mechanical ventilation. Data were collected prospectively during hospitalization and maintained in the NICU database. Early colostrum (administration < 24 hours of life) information was obtained retrospectively by chart review.

Descriptive statistics were used to describe neonatal and maternal demographics, neonatal interventions (medications, respiratory support), and outcomes for all three epochs. To compare differences between epochs, Kruskal-Wallis test was used for continuous variables while Chi-squared test and Fischer's exact test were used for categorical variables. Post hoc multiple comparison corrections included Dunn's test for continuous variables and Bonferroni correction for categorical variables. Multivariable logistic regression adjusting for GA was performed for all outcome measures. We also performed subgroup analysis in ELBW (< 1000 g) infants with outcomes adjusted for both GA and male sex. Regression model outliers were assessed with Pregibon's delta beta influence statistic. Specification link test was used to assess model specificity. Hosmer-Lemeshow test was used to assess goodness-of-fit. Variance inflation factor was used to assess each model for multicollinearity. We also present half yearly statistical process control (SPC) “p” charts for NICU intubation and percentage of VPI intubated in NICU in birth weight subgroups of < 1000 g, 1000-1499 g, and ≥ 1500 g. P values <0.05 were considered statistically significant. Statistical analysis was performed using STATA (StataCorp. 2015. Stata Statistical Software: Release 14. College Station, TX: StataCorp LP).

For benchmarking purposes, we presented temporal trends in a subgroup of VPI infants (400-1500 g birth weight or 22-31 weeks GA) in our center compared to other California NICUs participating in California Perinatal Quality Care Collaborative (CPQCC). In this high risk subgroup of VPI, we used control charts to track use of CPAP, NIMV, conventional mechanical ventilator, and high frequency ventilator. We also present the five-year cumulative (2013-2017) risk-adjusted outcomes of discharge home on oxygen and PMA at discharge in 211 consecutive inborns at our center. We compared these infants to all inborns (2014-2016) at 140 California NICUs, N=16,705, 28% of whom were cared for in 18 regional NICUs with level of care comparable to ours.

## 3. Results

### 3.1. Study Population

Of the 640 consecutive inborn VPI admitted to our NICU 2008-2017, 55% were < 1500 g at birth (VLBW) and 22% were < 1000 g at birth (ELBW).

### 3.2. Infant Demographics and Maternal Characteristics

Table 1 shows infant demographic and maternal characteristics for VPI and ELBW infants.

Across all epochs, VPI and ELBW were comparable for GA, BW, race, and vaginal birth. ELBW infants were 68% male in 2008-2010, significantly higher than 44% and 51% in the two subsequent epochs. Any maternal ANS use was > 94% and ANS use greater than 24 hours prior to delivery was > 73% in all three epochs. Increased prenatal magnesium use was noted in > 70% of VPI and > 80% ELBW in the third epoch compared to < 30% and < 40%, respectively, in the first epoch. Maternal antibiotic use was similar for ELBW infants (~50%) in all epochs. However, there was a decrease in VPI maternal antibiotic use from 57% in the first and second epoch to 46% in the third epoch.

Non-Hispanic white infants have higher risk of lung injury including pneumothorax and CLD [10]; we did not demonstrate differences in proportion of non-Hispanic whites across all epochs in the VPI and ELBW subgroup.

### 3.3. Respiratory Care and Pertinent NICU Practices

Table 2 shows respiratory and other pertinent NICU care processes. DR intubation risk declined significantly in VPI from 23% to 5% and in ELBW infants from 64% to 19% in the first and third epoch respectively. There was a significant reduction in the risk of invasive ventilation from a baseline of 37% to 17% in VPI and 92% to 51% in ELBW infants, but there was no reduction in the duration of ventilation for intubated VPI and ELBW infants. There was a significant increase in the number of days of CPAP use in VPI but not in ELBW. There was a sustained decrease in use of surfactant in the second and third epoch (2011-2017) in both VPI and ELBW. Caffeine use progressively increased for all VPI, while it remained high (85-97%) and unchanged throughout all three epochs in ELBW infants. There was a continued decrease in use of postnatal steroids and antibiotics both in VPI and ELBW infants. Colostrum use within the first 24 hours steadily increased in both VPI and ELBW infants.

### 3.4. Respiratory Outcomes

**Figure 2:**
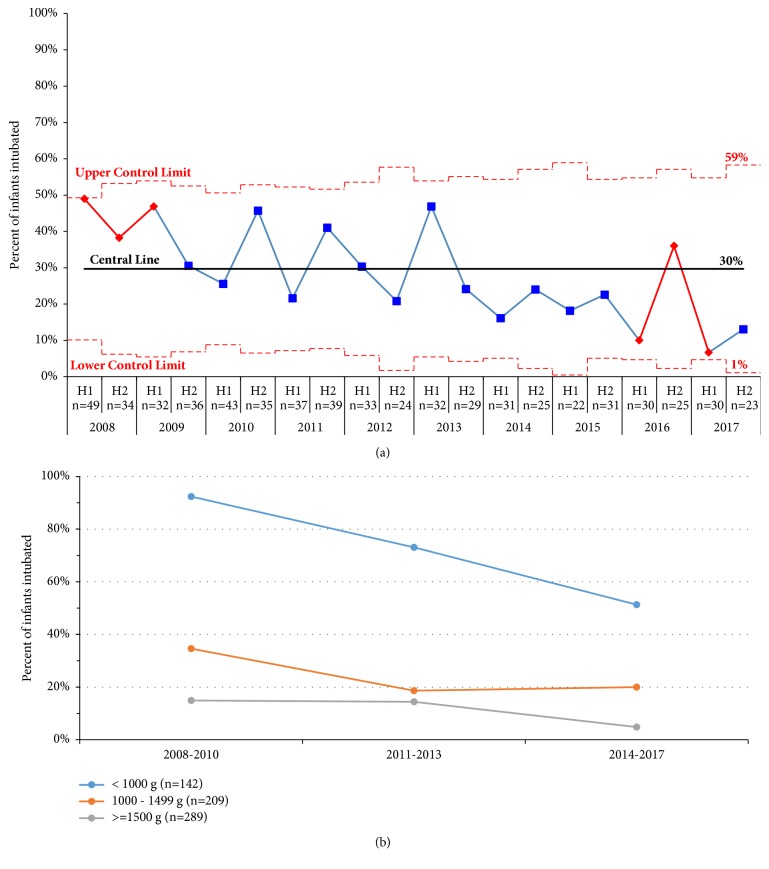
NICU intubation. (a) Ever intubation risk of < 33 week preterm infants illustrated by statistical process control half yearly “p” chart and (b) risk reduction in 3 exclusive subgroups over the 3 epochs.

Table 3 shows infant morbidity and mortality outcomes. Risk of intubation during NICU stay in VPI decreased from 39% to 18% and in ELBW infants, from 92% to 51% from the first to third epochs, respectively. This reduction in intubation remained significant even after adjusting for GA in VPI and adjusting for GA and male sex in ELBW infants.  Figure 2(a) uses SPC to illustrate the temporal decline in NICU intubation of VPI.  Figure 2(b) illustrates that the most significant decline in intubation occurred in ELBW infants, while 1000-1500 g infants maintained gains made earlier in the decade (2011-2013). There was a significant decrease in pneumothorax in ELBW infants from 17% in the first epoch to 0% in the third epoch that remained significant even after adjusting for GA and male sex. There was no significant difference in CLD.

There is a trend towards decrease in discharge home on oxygen in both VPI (from 5% to 2%) and ELBW infants (from 23% to 11%) from the first to third epoch, although not statistically significant.

### 3.5. Neonatal Mortality and Morbidity

There was no significant difference in mortality or major morbidities in VPI; however, we demonstrated a significant increase in survival without major morbidity in ELBW infants. There was an increase in the PMA at discharge in VPI, although not statistically significant after adjusting for GA. There was no increase in PMA at discharge in ELBW infants.

### 3.6. Benchmarks

**Figure 3:**
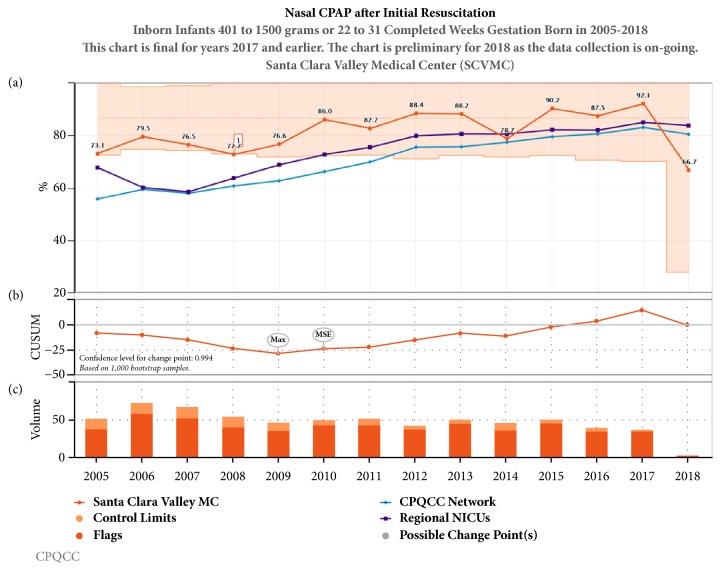
Annual trends in nasal CPAP after the initial resuscitation 2005-2017. (a) Unadjusted control chart with the orange shaded area representing the control limits and orange line representing the rate of CPAP use in our center. The purple line represents the 23 regional NICUs and the blue line represents all 140 NICUs in CPQCC (California Perinatal Quality Care Collaborative). (b) The CUSUM (Cumulative Sum Control) chart shows possible points of temporal change. (c) The volume bar shows the sample size and the event incidence that subdivides the bar.

**Figure 4:**
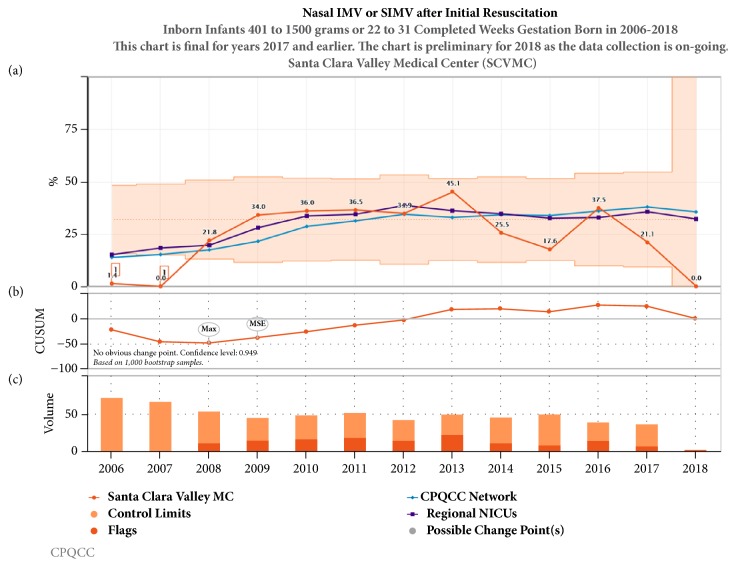
Annual trends in nasal IMV or SIMV after initial resuscitation 2005-2017. (a) Unadjusted control chart with the orange shaded area representing the control limits and orange line represent the rate of nasal IMV or SIMV use in our center. The purple line represents the 23 regional NICUs and the blue line represents all 140 NICUs in CPQCC (California Perinatal Quality Care Collaborative). (b) The CUSUM chart shows possible points of temporal change. (c) The volume bar shows the sample size and the event incidence that subdivides the bar.

**Figure 5:**
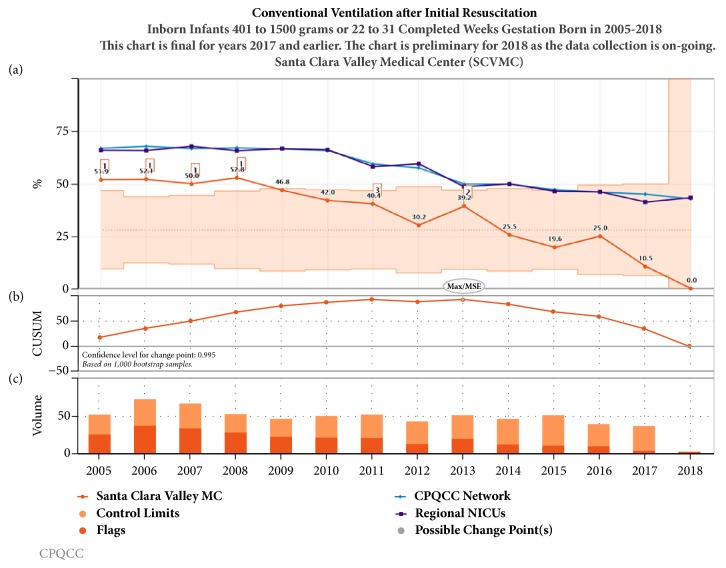
Annual trends in conventional ventilation after initial resuscitation 2005-2017. (a) Unadjusted control chart with the orange shaded area representing the control limits and orange line representing the rate of conventional ventilation use in our center. The purple line represents the 23 regional NICUs and the blue line represents all 140 NICUs in CPQCC (California Perinatal Quality Care Collaborative). (b) The CUSUM chart shows possible points of temporal change. (c) The volume bar shows the sample size and the event incidence that subdivides the bar.

**Figure 6:**
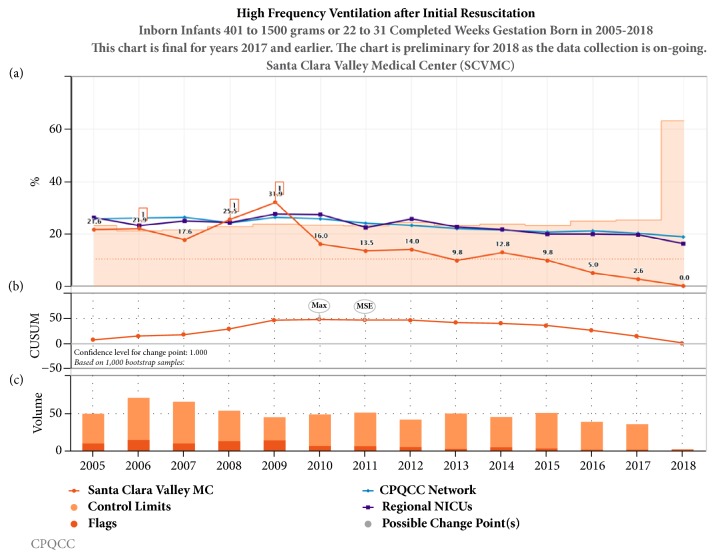
Annual trends in high frequency ventilation after initial resuscitation 2005-2017. (a) Unadjusted control chart with the orange shaded area representing the control limits and orange line representing the rate of high frequency ventilation use in our center. The purple line represents the 23 regional NICUs and the blue line represents all 140 NICUs in CPQCC (California Perinatal Quality Care Collaborative). (b) The CUSUM chart shows possible points of temporal change. (c) The volume bar shows the sample size and the event incidence that subdivides the bar.

**Figure 7:**
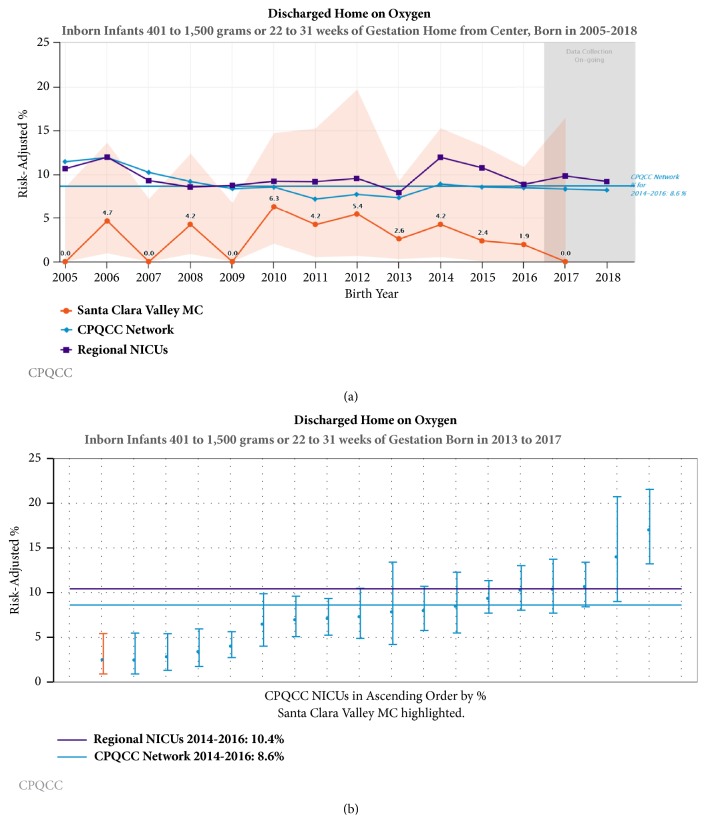
Risk-adjusted discharge home on oxygen rates for inborn infants at our center compared with other regional NICUs in California, 2013 to 2017. (a) The orange line with orange shaded upper and lower control limits represents our center. The purple line represents regional NICUs in CPQCC. The blue line represents all NICUs in the CPQCC network. (b) Each vertical line represents a regional center NICU participating in CPQCC. Dots represent the 5-year (2013 to 2017) aggregate risk-adjusted rate of discharge home on oxygen, and the vertical lines extend up to the 95% confidence limits for the risk-adjusted rate for each center. Our center is the red line. The horizontal reference line is the average rate of discharge home on oxygen for all regional NICUs in CPQCC.

**Figure 8:**
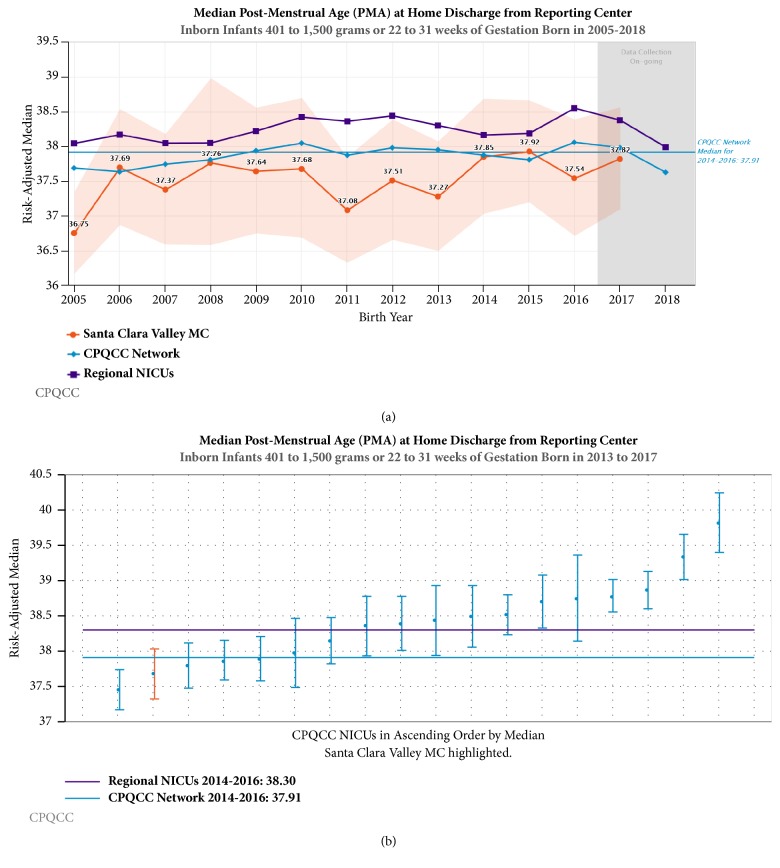
Risk-adjusted trends in median postmenstrual age (PMA) at home discharge for inborn infants, 2005-2017. (a) The orange line with orange shaded area between upper and lower control limits represents our center. The purple line represents 23 regional NICUs and the blue line represents all 140 NICUs in CPQCC (California Perinatal Quality Care Collaborative). (b) Each vertical line represents a regional center NICU participating in CPQCC. Dots represent the 5-year (2013 to 2017) aggregate risk-adjusted median PMA at discharge, and the vertical lines extend up to the 95% confidence limits for the risk-adjusted rate for each center. Our center is the red line. The horizontal reference line is the average rate of discharge home on oxygen for all regional NICUs in CPQCC.

Noninvasive strategies including CPAP and NIMV (Figures 3 and 4) have been increasing over the last decade in California, while conventional mechanical ventilation (Figure 5) and high frequency oscillatory ventilation (Figure 6) are decreasing. Despite this trend, for babies born <32 weeks and birth weight 401- 1500 g, discharge home on oxygen has remained stable in California NICUs at 8.6% (Figure 7) in our study decade 2008-2017. Our NICU, however, shows an inborn risk-adjusted discharge home on oxygen rate that is ~2.5% (2013-2017), compared to 10.4% (2014-2016) for inborn infants cared for in California regional NICUs. Our infants were also discharged at a comparable PMA if not sooner (Figure 8).

## 4. Discussion

With early initiation of noninvasive modes of ventilation in delivery room, we show a significant decrease in intubation and duration of invasive ventilation without worsening mortality or major neonatal morbidities for VPI in the three epochs studied. There is a suggestion of decrease in discharge home on oxygen, although not statistically significant. We also show improved survival without major morbidity in the smallest of these infants, the ELBW population.

### 4.1. Increasing CPAP and Decreasing Invasive Ventilation

Early CPAP in very preterm infants is associated with decreased number of days of invasive ventilation without any change in neonatal outcomes compared to early intubation [11, 12]. We have shown in an innovative clinical setting that there is a reduction in intubation accompanied by increase in CPAP days in VPI. We did not see any significant change in other neonatal mortality and morbidities in the VPI. However, in ELBW infants, there is a reduction in pneumothorax and increased survival without major morbidities. We adapted DR CPAP and bubble CPAP at the beginning our study period followed by standardization of multiple other noninvasive respiratory support (Figure 1). During the study period, compared to other regional NICUs across California, our center has shown lower risk of invasive ventilation (conventional and high frequency ventilation) with fewer infants discharged home on oxygen. These findings reassure us that commiting to noninvasive strategies does not lead to adverse pulmonary outcomes nor prolonged hospitalization.

### 4.2. Increasing Magnesium Use

Ever since the early report of rapid infusion of magnesium preventing intubation in two young adults with status asthmaticus in the emergency room [13], there has been growing interest in magnesium use in critical care settings [14]. There is also reassurance that prenatal magnesium use does not adversely affect cardiorespiratory status in preterm infants < 29 weeks GA in those exposed to antenatal magnesium compared with those without magnesium exposure [15]. Our current study reports a striking temporal increase in prenatal magnesium use in both VPI and ELBW infants accompanied by a temporal decline in intubation rates. There is a significant reduction in intubation over the epochs, even after adjusting for perinatal magnesium in VPI (adjusted odds ratio [adjOR] 0.53, p < 0.001) and ELBW (adjOR 0.33, p = 0.017). In the ELBW population, maternal magnesium appears to be associated with lower risk of intubation after adjusting for GA, male sex, and epoch (adjOR 0.05, p = 0.001). These findings require further validation.

### 4.3. Increasing Colostrum Use and Decreasing Neonatal Antibiotics

Optimal colonization of infant microbiota has been linked to improved outcome. Use of mothers' own milk has been associated with a reduction in CLD [16]. In our study we see a temporal increase in early introduction of colostrum with a decrease in exposure to any antibiotic while in the NICU. Whether these practices are related to the decline in intubation rates also requires further investigation.

### 4.4. Caffeine and Vitamin A

Caffeine [17, 18] and vitamin A [19] use have been associated with reduction in CLD. We report increased caffeine use in VPI infants during the study period, albeit no increase in vitamin A use. Our standardized criteria for initiating vitamin A in VPI included invasive ventilation at 72 hours of life. However, the reduction in invasive ventilation and intermittent periods of vitamin A unavailability led to lower use of vitamin A during the second epoch.

### 4.5. Minimally Invasive Methods of Surfactant Administration

Surfactant is beneficial in preterm infants with respiratory distress syndrome. However, the method of surfactant administration (via endotracheal tube insertion followed by invasive mechanical ventilation) is complicated by lung injury. Less invasive techniques such as INSURE (INtubate, SURfactant, Extubate) and LISA (Less Invasive Surfactant Administration) are methods that improve respiratory outcomes [20]. Decreased intubation in the last epoch may be attributable to the introduction of LISA into our practice in 2017.

### 4.6. Study Limitations and Future Directions in Clinical Practice

We are a single center presenting only inborn VPI experience at a highly resourced regional perinatal center and NICU. Hence the generalizability of our experience is limited to centers with similar populations and practices. We have a very high antenatal steroid exposure (> 93-96%) and DCC of at least 30-60 s in >83% of VPI. We have increasing early introduction of colostrum and decreasing antibiotic exposure in our very preterm population to promote favorable infant colonization. Another study limitation is our very low proportion of non-Hispanic white infants, a population known to have higher risk of respiratory morbidities including pneumothorax and CLD.

A decade is a relatively long time in modern medicine with major impact global clinical trials and studies attenuating clinical practice [11, 12, 21–23] in VPI. Future directions in minimizing lung injury include minimally invasive surfactant therapy (MIST). MIST (including administration of surfactant by intrapharyngeal instillation, nebulization, laryngeal mask, and thin catheter) allows for a spontaneously breathing infant to remain on CPAP but also benefit from surfactant therapy [24]. Developing a safe and effective mode of delivering MIST is critical since CPAP is the primary mode of respiratory support in preterm infants.

## 5. Conclusion

We demonstrate that early noninvasive cardiorespiratory support and minimizing DR intubation reduce the risk of NICU intubation without added risk of NICU morbidity. This is true in an era of increased caffeine and perinatal neuroprotective magnesium use and declining surfactant replacement therapy. Minimizing intubation and decreasing invasive ventilation are associated with decreased risk of discharge home on oxygen. This may also confer an advantage for survival without major morbidity for ELBW infants. Furthermore, perinatal magnesium may reduce risk of intubation in the ELBW infant. These findings require further validation and study. Reducing risk of home discharge on oxygen presents a compelling opportunity for further improvement in the care of the very preterm infant.

## Figures and Tables

**Table 1:** Infant demographics and maternal characteristics in very preterm and extremely low birth weight infants.

	**Very preterm infants, < 33 weeks gestation**				**Extremely low birth weight infants, < 1000 g**			
**Variable,**	**2008-2010**	**2011-2013**	**2014-2017**	***p***	**2008-2010**	**2011-2013**	**2014-2017**	***p***
%** or Median (IQR)**	**n** = 229	**n** = 194	**n** = 217		**n** = 53	**n** = 52	**n** = 37	
**Infant Demographics**								
Gestation, weeks	30.6 (27.7 - 32.0)	30.6 (27.6 - 31.9)	31.0 (28.9 - 32.0)	ns	25.6 (24.9 - 26.9)	26.4 (25.0 - 27.6)	25.6 (24.9 - 28.6)	ns
Birth weight, grams	1440 (1030 - 1750)	1383 (950 - 1720)	1450 (1140 - 1870)	ns	780 (630 - 880)	790 (700 - 895)	797 (720 - 920)	ns
Male	62	58	58	ns	68	44	51	0.045^*∗*^
Non-Hispanic white	10	14	11	ns	8	15	5	ns
Vaginal delivery	38	38	38	ns	15	35	22	ns
Multiple	20	18	19	ns	21	12	11	ns
Very low birth weight	56	58	52	ns	-	-	-	-
**Maternal Characteristics**								
Any antenatal steroids	97	97	94	ns	96	98	95	ns
Antenatal steroids > 24 h	73	75	75	ns	75	83	73	ns
Prenatal magnesium	26	56	74	<0.001^*∗*†‡^	38	65	81	<0.001^*∗*†^
Antibiotics	57	57	46	0.029^†‡^	49	56	46	ns

*∗*Statistically significant difference between 2008-2010 and 2011-2013.

†Statistically significant difference between 2008-2010 and 2014-2017.

‡Statistically significant difference between 2011-2013 and 2014-2017.

**Table 2:** Respiratory and pertinent NICU care processes.

	**Very preterm infants, < 33 weeks gestation**				**Extremely low birth weight infants, < 1000 g**			
**Variable,**	**2008-2010**	**2011-2013**	**2014-2017**	***p***	**2008-2010**	**2011-2013**	**2014-2017**	*** p***
%** or Median (IQR)**	**n** = 229	**n** = 194	**n** = 217		**n** = 53	**n** = 52	**n** = 37	
Delivery room intubation	23	15	5	<0.001^†‡^	64	40	19	<0.001^*∗*†^
Invasive ventilation	37	30	17	<0.001^†‡^	92	71	51	<0.001^*∗*†^
Invasive ventilation, days^§^	4 (1 - 20)	4 (1 - 13)	4.6 (2 - 16)	ns	18 (5 - 32)	9 (3 - 17)	15 (5 - 39)	ns
Non invasive mechanical ventilation (NIMV)	18	23	17	ns	58	65	65	ns
NIMV, days^§^	6 (3 - 15)	10 (2 - 17)	9 (2 - 24)	ns	8 (4 - 25)	10 (5 - 21)	19 (4 - 27)	ns
Continuous positive airway pressure (CPAP)	78	78	84	ns	89	83	81	ns
CPAP, days^§^	4 (2 - 19)	4 (2 - 16)	7 (2 - 21)	0.004^†^	22 (5 - 39)	25 (10 - 43)	30 (16 - 40)	ns
Surfactant	27	18	20	0.042^*∗*^	74	40	43	0.001^*∗*†^
Caffeine	48	58	69	<0.001^†‡^	85	94	97	ns
Vitamin A	15	1	13	<0.001^*∗*‡^	58	2	46	<0.001^*∗*‡^
Post natal steroids	10	7	3	0.01^†^	43	23	16	0.01^†^
Colostrum within 24 hours	45	76	86	<0.01^*∗*†‡^	38	72	84	<0.001^*∗*‡^
Infant antibiotics	67	59	41	<0.01^†‡^	96	90	73	0.004^†^

*∗*Statistically significant difference between 2008-2010 and 2011-2013.

†Statistically significant difference between 2008-2010 and 2014-2017.

‡Statistically significant difference between 2011-2013 and 2014-2017.

§Data summarized for those infants who received invasive ventilation, NIMV, CPAP, and nasal cannula.

**Table 3:** Respiratory outcomes, mortality, and morbidity.

	**Very preterm infants, < 33 weeks gestation (GA)**					**Extremely low birth weight infants, < 1000 g**				
**Variable,**	**2008-2010**	**2011-2013**	**2014-2017**	***p***	***p *adjusted**	**2008-2010**	**2011-2013**	**2014-2017**	***p***	***p *adjusted for**
%** or Median (IQR)**	**n** = 229	**n** = 194	**n** = 217		**for GA**	**n** = 53	**n** = 52	**n** = 37		**male sex and GA**
**Respiratory Outcomes**										
NICU intubation	39	31	18	<0.001^†‡^	<0.001	92	73	51	<0.001^*∗*†^	<0.001
Pneumothorax	5	3	2	ns	ns	17	10	0	0.01^†^	0.018
Chronic lung disease	12	13	14	ns	ns	50	46	43	ns	ns
Oxygen at discharge	5	3	2	ns	ns	23	10	11	ns	ns
**Mortality and Morbidity**										
Mortality	6	6	2	ns	ns	19	17	11	ns	ns
Major intraventricular hemorrhage	8	6	4	ns	ns	26	19	16	ns	ns
Nosocomial infection	8	5	4	ns	ns	26	15	16	ns	ns
Severe retinopathy of prematurity	6	3	4	ns	ns	25	12	24	ns	ns
Necrotizing enterocolitis (NEC)	5	5	2	ns	ns	19	13	8	ns	ns
NEC / Spontaneous Intestinal perforation	5	5	2	ns	ns	19	15	8	ns	ns
NICU length of stay, days	37 (21 - 63)	35 (22 - 61)	39 (24 - 61)	ns		101 (69 - 116)	86 (54 - 102)	87 (64 - 107)	ns	
Post menstrual age at discharge, weeks	35.9 (34.6 - 38.0)	35.9 (34.6 - 37.6)	36.1 (35.3 - 38.1)	0.008^†‡^		40.0 (35.9 - 42.1)	38.4 (35.7 - 40.9)	39.1 (37.7 - 42.1)	ns	
Survival without major morbidity	74	76	80	ns	ns	15	25	41	0.02^†^	0.021

*∗*Statistically significant difference between 2008-2010 and 2011-2013.

†Statistically significant difference between 2008-2010 and 2014-2017.

‡Statistically significant difference between 2011-2013 and 2014-2017.

## Data Availability

The data used to support the findings of this study are available from the corresponding author upon request.

## References

[1] Lee H. C., Powers R. J., Bennett M. V. (2014). Implementation methods for delivery room management: A quality improvement comparison study. *Pediatrics*.

[2] Elias N., OBrodovich H. (2006). Clearance of Fluid From Airspaces of Newborns and Infants. *NeoReviews*.

[3] Foglia E. E., Jensen E. A., Kirpalani H. (2017). Delivery room interventions to prevent bronchopulmonary dysplasia in extremely preterm infants. *Journal of Perinatology*.

[4] Moessinger A. C., Harding R., Adamson T. M., Singh M., Kiu G. T. (1990). Role of lung fluid volume in growth and maturation of the fetal sheep lung. *The Journal of Clinical Investigation*.

[5] Manani M., Jegatheesan P., DeSandre G., Song D., Showalter L., Govindaswami B. (2013). Elimination of admission hypothermia in preterm very low-birth-weight infants by standardization of delivery room management. *The Permanente Journal*.

[6] Song D., Jegatheesan P., DeSandre G., Govindaswami B. (2015). Duration of cord clamping and neonatal outcomes in very preterm infants. *PLoS ONE*.

[7] Lasswell S. M., Barfield W. D., Rochat R. W., Blackmon L. (2010). Perinatal regionalization for very low-birth-weight and very preterm infants a meta-analysis. *Journal of the American Medical Association*.

[8] Sherman M., Lauriello N., Aylward G. Follow-up of the NICU Patient. https://emedicine.medscape.com/article/1833812-overview#showall.

[9] Stoll B. J., Hansen N. I., Bell E. F. (2010). Neonatal outcomes of extremely preterm infants from the NICHD Neonatal Research Network. *Pediatrics*.

[10] Profit J., Gould J. B., Bennett M. (2017). Racial/Ethnic Disparity in NICU Quality of Care Delivery. *Pediatrics*.

[11] Morley C. J., Davis P. G., Doyle L. W., Brion L. P., Hascoet J.-M., Carlin J. B. (2008). Nasal CPAP or intubation at birth for very preterm infants. *The New England Journal of Medicine*.

[12] Finer N. N., Carlo W. A., Walsh M. C. (2010). Early CPAP versus Surfactant in Extremely Preterm Infants. *The New England Journal of Medicine*.

[13] Schiermeyer R. P., Finkelstein J. A. (1994). Rapid infusion of magnesium sulfate obviates need for intubation in status asthmaticus. *The American Journal of Emergency Medicine*.

[14] Panahi Y., Mojtahedzadeh M., Najafi A. (2017). The role of magnesium sulfate in the intensive care unit. *EXCLI Journal*.

[15] De Jesus L. C., Sood B. G., Shankaran S. (2015). Antenatal magnesium sulfate exposure and acute cardiorespiratory events in preterm infants. *American Journal of Obstetrics & Gynecology*.

[16] Dicky O., Ehlinger V., Montjaux N. (2017). Policy of feeding very preterm infants with their mother's own fresh expressed milk was associated with a reduced risk of bronchopulmonary dysplasia. *Acta Paediatrica*.

[17] Henderson-Smart D. J., De Paoli A. G. (2010). Methylxanthine treatment for apnoea in preterm infants. *Cochrane Database of Systematic Reviews*.

[18] Pakvasa M. A., Saroha V., Patel R. M. (2018). Optimizing Caffeine Use and Risk of Bronchopulmonary Dysplasia in Preterm Infants. A Systematic Review, Meta-analysis, and Application of Grading of Recommendations Assessment, Development, and Evaluation Methodology. *Clinics in Perinatology*.

[19] Darlow B. A., Graham P. J., Rojas-Reyes M. X. (2016). Vitamin A supplementation to prevent mortality and short- and long-term morbidity in very low birth weight infants. *Cochrane Database of Systematic Reviews*.

[20] Isayama T., Iwami H., McDonald S., Beyene J. (2016). Association of Noninvasive Ventilation Strategies With Mortality and Bronchopulmonary Dysplasia Among Preterm Infants. *Journal of the American Medical Association*.

[21] Askie L. M., Brocklehurst P., Darlow B. A., Finer N., Schmidt B., Tarnow-Mordi W. (2011). NeOProM: neonatal oxygenation prospective meta-analysis collaboration study protocol. *BMC Pediatrics*.

[22] Yoder B. A., Stoddard R. A., Li M., King J., Dirnberger D. R., Abbasi S. (2013). Heated, humidified high-flow nasal cannula versus nasal CPAP for respiratory support in neonates. *Pediatrics*.

[23] Abdel-Hady H., Shouman B., Aly H. (2011). Early weaning from CPAP to high flow nasal cannula in preterm infants is associated with prolonged oxygen requirement: A randomized controlled trial. *Early Human Development*.

[24] Shim G.-H. (2017). Update of minimally invasive surfactant therapy. *Korean Journal of Pediatrics*.

